# Elements of Effective Interventions for Addressing Intimate Partner Violence in Latina Women: A Systematic Review

**DOI:** 10.1371/journal.pone.0160518

**Published:** 2016-08-09

**Authors:** Carmen P. Alvarez, Patricia M. Davidson, Christina Fleming, Nancy E. Glass

**Affiliations:** Community-Public Health, Johns Hopkins University School of Nursing, Baltimore, Maryland, United States of America; Institute for Health & the Environment, UNITED STATES

## Abstract

**Background:**

Intimate partner violence remains a global problem and is of particular concern in Latina diasporas.

**Aim:**

To identify effective elements of interventions to address intimate partner violence in Latina women.

**Method:**

The systematic review was undertaken according to Preferred Reporting Items for Systematic Reviews and Meta-Analyses (PRISMA) guidelines. We focused the search on intervention studies assessing intimate partner violence as an outcome measure and on publications in English and Spanish from the last 11 years (2004–2015).

**Results:**

Despite the scope of the problem, from the 1,274 studies screened only four met the search criteria and only a single study included an exclusive Latino population. Of the four interventions, one was only as effective as the control treatment. Heterogeneity of study populations and designs prohibited meta-analytic methods.

**Conclusions:**

Theoretically derived interventions that are gender specific, culturally appropriate, target mutual aid through group dynamics, and that are developed collaboratively with the target population are likely to be most effective.

## Introduction

Intimate partner violence (IPV) defined as “behaviour by an intimate partner that causes physical, sexual, or psychological harm, including acts of physical aggression, sexual coercion, psychological abuse, and controlling behaviours” [1] remains a prevalent global health problem. Globally, an estimated 1 in 3 women will experience IPV and/or sexual violence [1]. Despite the growing recognition of violence against women, evidence to support best practices for responding to violence against women remains lacking [2, 3]. Further, questions remain as to whether sub-populations can access evidence-based IPV interventions and whether such interventions are effective in addressing IPV among these populations. In 2014, the 67^th^ World Health Assembly [4] called for health services to have a more impactful response to secondary prevention of IPV; however to meet this goal the gaps in understanding best practices for secondary prevention of IPV must be addressed.

High-income countries such as the United States (US) remain challenged with meeting health care needs of many of its underserved sub-populations, such as the Latina population. Latinas in the U.S, particularly immigrant Latinas, continue to have limited access to formal resources for IPV intervention. Examining which elements of interventions have demonstrated to decrease IPV among Latina women could inform health service responses in resource-constrained areas or high-income countries with growing immigrant populations, particularly Latina diaspora.

The Latino population is not only one of the fastest growing populations in the US (now constituting 18% of the US) but is also growing in Canada (National Household Survey, 2011), and increasingly Europe [5, 6]. The rapid growth of a young Latino population–particularly in the US [7] has drawn greater attention to IPV among Latina women. In the US, approximately a third of Latina women report a history of IPV [8]. Compared to non-Latina whites, Latina women are more likely to experience more severe adverse effects from IPV, such as depression low self-esteem, and physical ailments [9–11]. Not only the largest and fastest growing minority group in the US, the Latino population is also the youngest. Young adult Latinos comprise almost 20% of the general population of the 18–25 year age group–a historic proportion[7]. Further, compared to the majority white population, Latinos experience higher rates of adverse health outcomes often related to risk behaviors including unplanned pregnancy, sexually transmitted infections, and HIV infection—all of which have been shown to have IPV as a contributing factor [12]. Thus, this growing and vulnerable population warrants attention regarding interventions to promote healthy relationships and mitigate IPV.

Increasing women’s ability to plan for their safety in any setting—by assessing their risk and prioritizing safety strategies to reduce repeat victimization in violent intimate relationships—is important to increase safety and improve health outcomes.

Suggested contributors to IPV among Latinos include the following: immigration status, lower socioeconomic status, acculturation stress, and *machismo—*a cultural term used in Latin America to describe male domination in the relationship[13, 14]. These factors not only serve as risk factors for IPV, but also as barriers for women safely leaving their abusive partners. For example, qualitative studies have revealed that some Latinas hesitate leaving their abusers in keeping with traditional norms of staying in a marriage [13–15] and, among undocumented women, for fear that reporting their abuser would result in their own deportation [14]. In cases when women were ready to report the IPV to an authority or leave the relationship, English literacy and understanding the social, health, and legal systems can serve as significant barriers for safety. Researchers also suggest that Latinas who are less acculturated to the United States may be more likely to internalize negative emotions related to their abuse because of the cultural expectation to not express anger [10]. As such, Latinas (especially monolingual Spanish speakers) are more likely to negate the severity of abuse, less likely to use formal services to address IPV, and when they do eventually use formal services, it is after extended periods of experiencing abuse [11, 16].

Developing linguistically and culturally appropriate interventions geared towards empowering Latinas in decision-making about their safety as well as seeking help from informal and formal systems is a high priority. In an effort to develop an appropriate intervention for Latina survivors of IPV, Davila and colleagues learned from participants what the ideal intervention would entail [17]. Their suggestions include: (1) having an intervention that increased their knowledge about women’s health as well as IPV, (2) using talking circles and interactive activities to enhance learning and keep participants engaged, and (3) hosting sessions in locations that would not make their partners suspicious (i.e., participants suggested that hosting the interventions at churches or schools would be preferred over a clinic).

The aim of this review was to identify elements of successful interventions to address IPV among Latina women. This review highlights promising and best practices for IPV interventions among Latinas, and identifies critical areas for further research.

## Method

### Search Method

The systematic review was undertaken according to Preferred Reporting Items for Systematic Reviews and Meta-Analyses (PRISMA) guidelines [18]. An informationist developed and conducted the search strategy with guidance and input from the lead author. The following search strategy was applied to PubMed, Embase, Cochrane, CINAHL, Scopus, and Web of Science: ("Domestic Violence"[Mesh] OR "domestic violence"[all] OR "domestic partner violence"[all] OR "family violence"[all] OR "dating violence"[all] OR "battered woman"[all] OR "battered wife"[all] OR "battered women"[all] OR "wife beating"[all] OR "partner violence"[all] OR "marital rape"[all] OR "intimate partner violence"[all] OR "partner abuse"[all] OR "spouse abuse"[all] OR "spousal abuse"[all]) AND ("Hispanic Americans"[Mesh] OR "Mexican Americans"[mesh] OR "Hispanic American"[all] OR "Hispanic Americans"[all] OR "Spanish American"[all] OR "Spanish Americans"[all] OR "Puerto Rican"[all] OR "Puerto Ricans"[all] OR "Latina"[all] OR "Latinas"[all] OR "Latino"[all] OR "Latinos"[all] OR "Cuban American"[all] OR "Cuban Americans"[all] OR "Hispanic"[all] OR "Hispanics"[all] OR "Mexican American"[all] OR "Mexican Americans"[all])). The search was limited to publications in English and Spanish from the last 11 years (2004–2015). The literature search and data analysis were performed in May 2015 by the study authors. The rationale for restricting the time window was to focus on contemporaneous immigration patterns and sociocultural aspects.

### Inclusion Criteria

The goal in reviewing the articles was to identify elements of promising or best practices for interventions that focused on prevention or response to IPV among Latina women. Articles were included in the review if they met the following criteria: (1) an intervention was described and tested, (2) had a sample size that included at least 25% Latinos–particularly Latina women, (3) the sample included subjects between 18–25 years of age, (4) measured intimate partner violence, and (5) the intervention had a positive outcome on intimate partner violence. The authors (C.A. & C.F.) independently reviewed the search results to determine whether the articles met the inclusion criteria; findings and differences were discussed and resolved.

### Quality Assessment of Intervention Studies

The first and senior author assessed the quality of the studies using “the Cochrane Collaboration’s tool for assessing risk of bias” [19]. This is a widely used tool to evaluate the quality and validity of intervention studies. Domains of this tool included evaluation for randomization, allocation concealment, blinding of participants and data collectors, attrition bias, and other potential threats to validity such as early cessation of the study or unbalanced baseline data. Using this tool, studies were characterized as having low, unclear, or high risk of bias. A study was classified as low-risk if any bias was unlikely to significantly alter the findings, unclear risk described studies in which the bias raised doubt about the findings, and high-risk studies consisted of methods that weakened confidence in the results.

## Results

**Fig 1:**
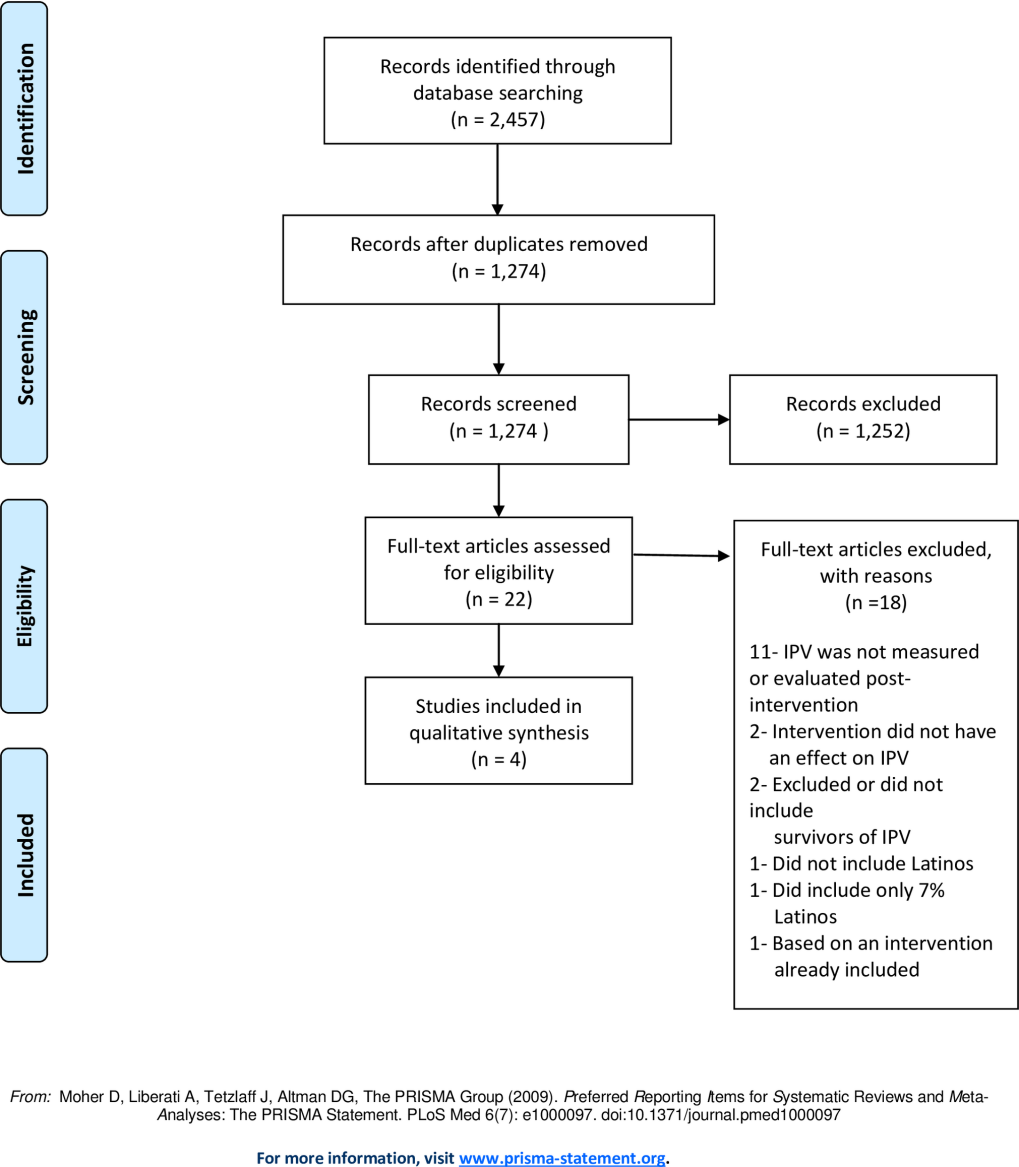
PRISMA Flowchart for selection of eligible research articles for this systematic review of effective interventions for decreasing intimate partner violence (IPV) among Latina survivors.

The literature largely addresses exploratory studies and predictors of IPV among Latino populations. A total of 1,132 articles were identified (Fig 1), while 4 met the study inclusion criteria [20–23]. The majority of the articles were excluded because there was no intervention and no measure of IPV. The selected study characteristics are presented in Table 1.

**Table 1:** Overview of Interventions in Systematic Review of Effective Interventions for Decreasing Intimate Partner Violence among Latina Women.

Author	Intervention & Aim	Setting & Population	Theoretical Framework & Intervention Description	Improved Violence Outcomes	Quality & Bias
McFarlane et al., 2006	Nurse-led intervention for secondary prevention for IPV	Settings: 1. Urban areas; 2. Primary care public health clinics; 3. Women Infants & Children clinics	Dutton’s Empowerment Model—Designed to empower survivors of IPV to engage in safety behaviors	Intervention & Control: 1.↓Threats of assault; 2.↓Assault; 3. ↓Risk for homicide; 4.↓Work harassment; 5.↓Safety behaviors	1. Powered sample size (N = 360); 2. Randomization; 3. No blinding; 4. Fidelity to intervention not evaluated; 5.Contamination not addressed; 6. 89% Retention; 7. Bias: Unclear risk
		Inclusion criteria: 1.18–44 years old; 2. English or Spanish speaking	Intervention included a March of Dimes brochure w/ 15-item safety plan; nurse counseling including anticipatory guidance, guided referrals, and supportive care		
		Population: 1. Mean age– 30; 2. 59.6% Latina; 3. 50% foreign-born; 4. Low income; 5. Low education	Intensity & Duration: One time 20 minute session		
Olds el at., 2004	Nurse and paraprofessionals conducting home visits to examine whether home visits provided by paraprofessionals would be as effective as those delivered by nurses	Settings: 1. Denver, Colorado; 2. Participants’ homes	Paraprofessionals and nurses worked with mothers to improve and engage in more health-related behaviors—enhancing parenting skills, thinking about family planning and work-life, and improving relationships with family and friends	Nurse-visited mothers: ↓Domestic violence	1.Powered sample size (N = 690); 2. Randomization; 3. Blinding; 4. Fidelity checks; 5. 80% retention; 6. Bias: Low risk
		Follow-up participants 2 years after the end of the intervention; Medicaid eligible, pregnant women expecting their first live birth	Intensity & Duration: Home visits during pregnancy and the first 2 years child’s life		
		47% Mexican-American			
Peragallo et al., 2012	*Salud Educacion Protecion y Autocuidado* (SEPA) Health, Education, Protection and Self-care to improve health and sexual health behaviors	Community based	Social-Cognitive Theory	Intervention group: ↓IPV over one year	1.Powered sample size (N = 548); 2. Randomization; 3. No blinding; 4. Fidelity checks; 5. 73% Retention; 6. Bias: Unclear risk
		Miami-Dade and Broward county, Florida	Sessions covered HIV/AIDS in the Hispanic community, STIs, HIV/AIDS prevention (e.g., condom use), negotiation and communication with the partner, IPV and substance abuse. Groups took place in community sites easily accessible to participants. Role play, participatory sessions, videos and discussions were used to build skills		
		Inclusion criteria: 1. Latina; 2. 18–50 years old; 3. Reported sexual activity in the last 3 months	Intensity & Duration: 5 2-hr sessions		
		Mean age: 38 year old			
Wray et al., 2013	Mutual-violence intervention to decrease victimization and perpetration of IPV between couples	No location provided	The intervention included sessions about: relationship skills, emotional awareness skills, and parenting/co-parenting skills	Intervention group: Men: ↓perpetration of physical assault, injury from female partner. Women: ↓physical assault and injury from male partner.	1.Pilot study; 2. No randomization; 3. Fidelity checks; 4. Bias: High risk
		Inclusion criteria: 1. Court-mandated to treatment for IPV/DV; 2. English-proficient and co-parent to at least one child	Intensity & Duration: 12 90- minute sessions	Recidivism rate: 4% for intervention couples; 50% for control.	
		Populations: Males: Mean age: 29, 65% Latino. Females: Mean age: 27, 55% Latina			

### Settings and Target Populations

All studies were conducted in the US and based on convenience samples. Participants were recruited from urban, primary care public health, and Women, Infants and Children (WIC) Program clinics [20], prenatal clinics [21], the general community [22], and from a court-mandated treatment program [23]. The interventions’ target populations included: mutually violent couples with a child, expectant and current mothers, female survivors of IPV, and sexually active Latina women. Two interventions were designed specifically to target IPV; the other interventions were designed to address other health behaviors, such as sexual risk behaviors, and maternal-child interaction, but also evaluated IPV. Interventions were delivered within a clinic setting, in participants’ homes, and in community areas.

All studies included a large sample of Latinos. One study included an exclusive Latino population, of which the majority were Spanish dominant or not acculturated to the US [22]. In the other studies, Latinos comprised 47%–65% of the study sample. Three studies were explicit about including both English or Spanish-speaking participants [20–22]. In one study, approximately 6% of the sample were monolingual Spanish speakers; however, there was no detail provided about how the instruments and intervention were tailored and delivered for this unique subset [21].

### Study Characteristics

Three of the interventions were tested with a randomized control trial design [20–22] and one pilot study was conducted with a quasi-experimental design [23]. Deliverers of the intervention included trained paraprofessionals [21], nurses [20, 21], and psychology doctoral students or master’s level clinicians [23]. One study described their facilitators in terms of educational level but did not provide their profession or background [22]. One study reported blinding data collectors to intervention or control group [21]. The other studies did not report blinding to the treatment groups, and those delivering the intervention also administered the questionnaires [20, 22, 23]. One study used public records to obtain conviction data for perpetrators of IPV [23], while the others obtained their outcome data only from self-report.

For the randomized-control trials (RCTs), a computer-generated process was used to randomize participants [20–22]. The duration of the interventions ranged from a one-time session lasting 20 minutes [20], to 12, 90-minute sessions [23]. Post-intervention follow-up ranged from 3-months to 2 years. The study using home-visits conducted assessments at 28 and 36 weeks gestational age, and during the first 4 years of the child’s life at 6, 12, 15, 21, 24, and 48 months.

### Intervention effects on IPV

McFarlane and others tested the effects of a nurse-led intervention on report of IPV among survivors of IPV [20]. Two years after a 20-minute nurse-led intervention or receipt of a referral card for women who screened positive for IPV, McFarlane and colleagues (2006) found that both groups of women–those in the intervention and control groups–reported greater safety behaviors, and reduced risk for lethal violence and work harassment. The intervention included receipt of a March of Dimes brochure with safety plan tips. The nurse also provided anticipatory guidance for leaving the abusive relationship, anticipated referrals (housing assistance, immigration needs), and provided psychosocial support. The control group received a wallet-sized card with community resources. In this study both forms of intervention–the referral card and safety plan with nurse counseling–were effective in reducing multiple forms of violence. The authors of this study did not address potential weaknesses such as fidelity or how they may have handled issues of contamination.

Olds et al. (2004) tested the effectiveness of delivering an intervention for improving maternal and child health outcomes with paraprofessionals versus nurses. During the home visits, nurses and paraprofessionals focused on health-promoting behaviors that could impact the pregnancy and child, as well as on improving mothers’ relationships with family and friends in order to establish a form of social support for the mother and child. At the four-year follow-up, mothers who received home visits from nurses, compared to the usual care, were less likely to report IPV. There was no effect on IPV for mothers visited by paraprofessionals compared to the usual care group. The researchers did not report comparisons between the paraprofessionals and nurses.

Peragallo and colleagues (2012) developed an intervention: *Salud*, *Educacion*, *Prevencion*, *Autocuidado (SEPA)* (Health, Education, Prevention, Self-care). The main objective of SEPA was to decrease sexual risk behaviors among Latina women. Although experiencing IPV was not a criterion for participating in the study, the descriptive analyses revealed that more than half the women in both groups (61% in the control and 67% in the intervention group) screened positive for IPV. The intervention consisted of 5 small group 2-hour sessions about HIV/AIDS among Latinos, sexual communication with partners, substance abuse, and IPV. The control group received all the intervention information in a one-time session (there was no report on duration of this session). At one-year follow-up, women from the intervention group were significantly less like to report IPV.

Wray and colleagues piloted an intervention targeting mutually violent couples [23]. Couples in the study were mandated by the court to attend either the intervention or a similar program within the community; participation in the intervention as a couple was not required. The 12-session intervention focused on building positive relationship behaviors such as affection, reducing negative behaviors such as criticizing, and promoting safe behaviors such as safety planning. Of the intervention participants who completed the conflict tactics scale (75.8% men and 64.3% women), men reported less perpetration of violence, and less injury inflicted from their female partner; the same pattern of findings was noted among women. When comparing rates of recidivism between the groups, those who did not participate in the intervention had the highest rates: 50% versus 4% among couples where both parties attended.

## Discussion

This review has identified that in spite of the magnitude of IPV as a public health problem and documentation of the problem within the diverse Latino population, prevention and response interventions are sparse. Our systematic review for effective interventions for Latinos retrieved only four interventions. Only one study focused exclusively on a Latina population [22], one study targeted survivors of IPV [20], and one focused on the couple dyad involved in the IPV [23]. We did not identify any interventions with positive outcomes for IPV prevention specifically targeting Latina survivors of IPV.

Among the four studies, there was heterogeneity in the quality of the studies, setting, content, and delivery of the intervention precluding meta-analysis. Given such variation, it is challenging to make comparisons related to essential elements for promising or best practices for IPV prevention and response interventions with Latinos. Nonetheless, there were some commonalities in the three interventions that reported reduced IPV in the intervention groups compared to control group. These three studies were all grounded in a theoretical framework [21–23], and involved multiple sessions; for example, two of the studies delivered the intervention in a group setting [22, 23]. Indeed, group sessions have been shown to increase self-awareness, feelings of empowerment, and also create a form of peer support [24, 25]. According to social cognitive theory, improvements in self-awareness, empowerment, peer support, and self-efficacy are all critical elements for behavior modification [26, 27].

Another commonality between the interventions was a focus on improving relationships with one’s partner and/or family members [21–23]. The SEPA intervention in particular focused on increasing sexual communication between couples[22]. The researchers of SEPA recently conducted analyses to evaluate whether partner communication contributes to reductions in IPV [28]. Their findings revealed that greater partner communication was associated with less male-to-female IPV. In other words, the potential “mechanism of action” of the intervention was the emphasis on healthy communication strategies. Participants in the other study interventions [21, 23] may have also learned about healthy communication and strategies to reduce conflict and thereby IPV.

Even though McFarlane and colleagues (2006) found that the 20-minute nurse intervention was almost as effective as providing a referral card, this finding is still useful. In this study both the control and intervention group received information about community resources. In a study that explored differences between women who sought formal services and those who did not, the main finding among the Latina women who did seek services was that they were informed about the available bilingual services for survivors of IPV [16]. Some of these women remarked that if no one had ever told them about such social services they never would have known and perhaps never have left the abusive relationship. In essence, in some cases, merely informing survivors of IPV about available resources can be an intervention.

Only one study was explicit about the effects of the intervention on different types of IPV [20]. Of the studies that used the CTS to measure IPV, one used the physical abuse items only [22] and the other two did not parse out how the interventions may have affected different types of IPV. When considering safety and psychological well-being of the participants, it would be important to know whether an intervention has an effect on diverse forms of IPV, including physical and sexual IPV and psychological abuse. Such a finding would have important implications for modifying the intervention as appropriate. For example, psychological abuse not only has adverse mental health effects, this type of IPV is also less likely to be reported [29] and may be more difficult to address through safety planning and other intervention strategies [30].

Although all of these studies included large proportions of Latinos, none of the investigators discussed how the intervention was tailored for the population. Only two studies described how they accommodated non-English-speaking participants [20, 22]; this included providing written materials in Spanish; however, there was no mention of including specific cultural values/practices in the intervention; for example, *familismo* (the belief of loyalty and respect for the family, and considering the family when making decisions about one’s life) [31]. This concept has been shown to influence how survivors of IPV make decisions about their relationship and safety, such as help-seeking behavior and confiding in family or friends [16]. Including such cultural values may have furthered our understanding about how including these values could yield more positive outcomes.

The level of acculturation in relation to effects of the intervention was also not explored in these studies. Acculturation was measured in two of the studies only to describe the sample [20, 22]. Given that the evidence that the level of acculturation is associated with IPV perpetration and safety strategies used by survivors [10, 32], it would be useful to know whether certain elements of an intervention are more appropriate with high or low-acculturated Latinas. The studies that included both Latinos and non-Latinos did not disaggregate the data to show whether there were any interaction effects of treatment and acculturation on IPV outcomes, such as reduction in physical or sexual violence.

None of these studies included bystanders or family members in the intervention. Although the study by Olds allowed for involvement of family members, the consistency of family inclusion and effect on IPV was not addressed [21]. Women’s perception of how family and community respond to IPV influences their help-seeking behaviors. At the same time, community members and family sometimes opt to “not get involved” in helping survivors out of concern for their own safety, belief that the survivor is at fault, and also because they are unaware of how to help [33]. Influencing social norms about IPV will be a critical component of primary and secondary prevention of IPV within the Latina community.

Despite the growing body of literature documenting the predictors, complexity, and survivor experiences of IPV among Latinos [10], evidence on how to address the problem is lacking. There are likely many challenges both researchers and community members may need to overcome in order to develop effective prevention and response interventions with Latinos. For example, in developing a culturally tailored intervention for sexual risk behavior, researchers encountered myths about why Latinos would not participate in such interventions, such as “Latinos won’t wear condoms because they are too macho” [34]. Similarly, when community members and service providers were interviewed about barriers to IPV interventions, they also reported “cultural barriers,” such as acceptance of IPV and desire to keep the family together [33]. At the same time, studies have demonstrated that these factors need not be considered as barriers but rather “cultural values” to include in intervention content [25, 34].

Using expert recommendations [35], the development of effective interventions for Latina IPV survivors will require a collaborative process. Coproduction of interventions by researchers with community members and survivors will facilitate better understanding of the problem, as well as how to work with “cultural barriers.” Building on Davila’s work [17] and others who have explored survivors’ suggestions for IPV interventions [29, 36], collaborative intervention development, implementation, and evaluation will most likely result in a community-driven, culturally, and linguistically appropriate intervention for the target population.

While this review focused on a Latina population in the US, our findings remain relevant for other immigrant populations across the globe. Developing interventions for IPV survivors that facilitate overcoming barriers such as language, culture, and family values are relevant to the many migrant populations seeking services for any health issue, including one as complex as IPV. Theory-driven and evidence-based solutions are critical to developing sustainable programs and long-term impacts for secondary prevention of IPV regardless of the setting in which it occurs.

Regardless of the target population, interventions guided by theory have been demonstrated to be more effective than those without a theoretical foundation [27]. Theoretical frameworks are tested models that can help the researcher understand antecedent and moderating factors and consider concepts that may be relevant to the phenomenon of interest. The use of theoretical frameworks can help researchers approach a problem with a more comprehensive perspective and best identify points of intervention. Interventions are more likely to be successful if they are developed in partnership with the target population, those who understand the context of the problem, and use behavioral change strategies that align with the norms of the target population [27, 35]. In other words, tailored and culturally appropriate interventions are most successful for changing health behaviors [37].

### Limitations

Our review found few interventions that met the inclusion criteria and only one that reported reducing IPV among Latino participants within the last 10 years. Given our goal of identifying effective interventions with Latina survivors of IPV, we limited our search to studies that had demonstrated a positive effect: reduction of IPV. We focused on the last 10 years to focus on current literature and with the anticipation that the literature about Latina survivors of IPV would parallel the growth of the Latino immigrant population in last decade; thus, we may have missed earlier effective interventions. Further, we targeted our search on survivors of IPV; it is possible that we may have also missed other interventions that targeted other issues, such as HIV and reproductive health for example, among Latinas and reported a reduction in IPV.

In spite of these limitations, this study has several strengths. First the study design allowed for Spanish language studies; second, the data extraction process and analysis was systematic and rigorous with consideration of gender and culturally specific dimensions of dealing with IPV.

### Recommendations for Future Research

Interventions targeting IPV are inherently multifaceted, involving complex sociocultural dimensions. The stressors of migration and commonly undocumented status exacerbate these challenges. Therefore issues of access need to be carefully deliberated to first not only achieve access but also negate unintended consequences of interventions. Therefore, considering issues pertaining to access are important. Although these aspects are addressed in study designs in the literature, the systematic documentation is not apparent. Adherence with reporting recommendations, such as the CONSORT statement for non-pharmacological interventions, will both increase the rigor of the studies and also assist in reporting the intervention to enable replication and testing [38].

In order to assess the applicability of future interventions, considering the following broad demands of access provided by Perchansky and Thomas [39] may be of use: (1) **Availability:** the volume of health resources fit with the volume and type of user need; (2) **Affordability:** the costs of services fit with users’ income and ability to pay; (3) **Accessibility:** the location of supply fits with the location of clients; (4) **Accommodation:** the organization of health care fits the clients’ demands; (5) **Acceptability:** the characteristics of the health service fit with the users’ attitudes and characteristics.

To address the significant problem of IPV, we require interventions that are not only efficacious but effective. Considering these factors in intervention development and design are important. Based upon the findings of this review, there are urgent needs to move from merely describing IPV to intervening to address the problem with profound health and social impacts. Achieving a standardized taxonomy for intervention description and development may enhance not only the development of interventions but the translation of successful interventions to usual care [40].

Future interventions should also target young adult populations (18–25). Young adults are disproportionately affected by IPV, yet none of the interventions we found targeted this specific age group. The transitional phase from adolescents to adulthood increases young adults’ vulnerability to engaging in health-risk behaviors and being at risk for IPV. Prevention and response interventions are needed before and during this transitional phase and high-risk age group.

## Conclusion

In spite of the documentation of the burden of IPV in Latino populations, solutions are less apparent and, as this diaspora increases in the US, effective interventions are critically needed. This review emphasizes the importance of developing and implementing interventions that are tailored and targeted to the needs of the Latino population. Targeting theoretically derived interventions that are gender specific, culturally appropriate, enhance mutual support through group dynamics, and that are developed collaboratively with the target population are likely to be most effective.

## Supporting Information

S1 PRISMA Checklist PRISMA 2009 Checklist. (DOC)
